# Gatekeepers of pancreas: TEAD and YAP

**DOI:** 10.18632/oncotarget.4607

**Published:** 2015-06-23

**Authors:** Santiago A. Rodríguez-Seguí, José Bessa

**Affiliations:** Laboratorio de Fisiología y Biología Molecular, Departamento de Fisiología, Biología Molecular y Celular, IFIBYNE-CONICET, Facultad de Ciencias Exactas y Naturales, Universidad de Buenos Aires, Buenos Aires, Argentina; Instituto de Biologia Molecular e Celular (IBMC), and Instituto de Investigação e Inovação em Saúde, Universidade do Porto, Porto, Portugal

The pancreas hosts some of the most debilitating and deadly diseases, including pancreatic cancer and diabetes mellitus. In autoimmune diabetes, for example, there is a massive destruction of the insulin producing cells of the pancreas. Pancreatic developmental defects can also result in a deficit of this cell type. To revert these important pancreatic diseases, researchers are currently trying to artificially generate insulin producing beta-cells for implantation and, in this way, suppress insulin administration [1]. However, to derive cells that function properly it is necessary to understand in depth the instructions that our body uses to produce them during fetal development. On the other hand, regarding pancreatic cancer, it is known that tumor cells very often recover characteristics similar to those of embryonic cells [2]. These are clear examples of the relevance to understand the identity of pancreatic progenitor cells.

The human genome contains the instructions to generate all the cell types that are formed during embryonic development. Building such complex systems is in part possible because the transcription of different sets of genes is sequentially activated or repressed generating cell-specific combinatorial profiles. Enhancers are one class of non-coding cis-regulatory sequences that control the transcription of genes. Also, the specific temporal and spatial regulatory activity of each enhancer is defined by clusters of transcription factors (TFs) that are bound to these sequences. Therefore, understanding the identity of pancreatic progenitor cells requires the identification of these regulatory sequences and the TFs that bind to them.

Our recent work published in Nature Cell Biology characterized the identity of human pancreatic progenitor cells [3]. For this purpose, we used human fetal pancreas, which was dissected from embryos at an early stage of gestation (~ 6 weeks), as well as pancreatic progenitor cells derived *in vitro* from human embryonic stem cells (hESC), that matched the developmental stage of cells in the fetal pancreas. We took advantage of high throughput sequencing techniques to identify genes that are selectively expressed in both samples and to produce a map of the genomic sequences that act as enhancers in the human embryonic pancreas. The results showed that pancreatic progenitor cells derived *in vitro* effectively recapitulate the main properties of the transcriptional program of the fetal progenitor cells, validating the *in vitro* system. In addition, further analysis of the regulatory regions active in pancreatic progenitor cells uncovered how genes can be transcriptionally regulated at this specific cell stage.

**Figure 1:**
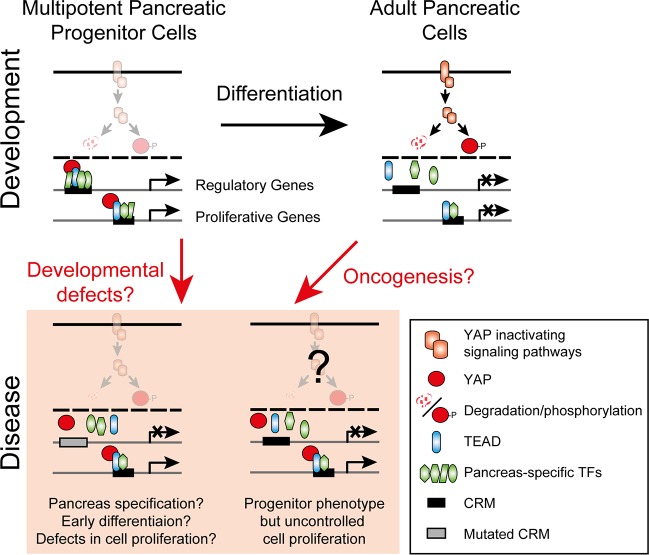
The role of YAP and TEAD in the activation of enhancers of pancreatic multipotent progenitor cells and their possible contribution to human pancreatic diseases Cis-regulatory modules (CRMs) are regions within the enhancers that show extensive TF co-binding.

We report that these regulatory regions are recognized by several TFs that have been previously described to be mutated in patients with congenital defects in pancreas development. Additionally, we found that the majority of these enhancers are recognized by TEAD proteins and its co-activator factor YAP, two important effectors of the Hippo signaling pathway [4]. Two recent reports have shown that pancreas-specific disruption of the upstream Hippo kinases Mst1/2 leads to acinar differentiation defects and changes in pancreas architecture [5, 6]. These reports, however, do not address whether Hippo signaling or TEAD are important for pancreatic progenitors. The results reported in our recent work show that TEAD and YAP play a key role in the identity of pancreatic progenitor cells. This was confirmed by functional studies, performed in progenitors derived from hESC, pancreatic bud explants from mouse embryos, and zebrafish, which showed that TEAD and YAP not only play a key role in the activation of these regulatory regions, but also control cell proliferation in embryonic pancreatic progenitor cells. We end up with a model for early pancreas development in which specific non-coding regions of the genome are exquisitely defined by co-binding of several pancreas-specific TFs, which are timely co-expressed at the progenitor cell stage (green shapes in Figure 1). A subset of such regions is also bound by TEAD proteins (light blue in Figure 1), and this combination of factors somehow marks them as “poised” enhancers, awaiting timely enhancer activation. Proper activation is provided by YAP nuclear translocation and binding to TEAD-targeted enhancers (red circles in Figure 1), thus leading to increased expression of target genes. Given that the control of YAP intracellular localization is mainly driven by the Hippo kinase cascade (orange shapes in Figure 1), this would confer a means for pancreatic progenitors to integrate extracellular signaling cues to control a timely cell differentiation program. The identified role for TEAD and YAP in this process opens up new research avenues towards disease development in humans. YAP has been described as an oncogene involved in several types of cancer, including pancreatic ductal adenocarcinoma [4]. Our results suggest that the reactivation of the pancreatic embryonic program in adult pancreatic cells could contribute to dedifferentiation and uncontrolled growth during pancreatic carcinogenesis. In this context, YAP, TEAD, or their pancreatic-specific downstream genes could be used as targets of new anti-cancer drugs. On the other hand, the regulatory program described in our work could be potentially exploited to control the growth and differentiation of *in vitro* derived pancreatic beta cells, aiming for treatment of diabetic patients in which these cells have been destroyed. Diabetes can also result from the impairment of proper beta-cell embryonic development, which has been classically attributed as the result of coding mutations in key pancreatic developmental genes. Using a preliminary version of the regulatory regions that we recently reported, the groups of Prof. Ferrer and Prof. Hattersley have shown that, in addition to coding mutations, regulatory mutations might also cause severe developmental defects of the pancreas, such as pancreatic agenesis [7]. Therefore, the atlas of regulatory elements of pancreatic progenitor cells described in our work could be used to define genomic hotspots of mutations with the potential to impair proper pancreas development. Indeed, with current technologies allowing the sequencing of a patient's genome at an affordable cost, this will become a possibility in the near future.

In summary, we have now unveiled a novel role for YAP and TEAD proteins in the control of gene expression during pancreas development. These findings now turn the spotlight on YAP and the Hippo pathway, placing them at the crossroads of pancreas formation. Special attention should be directed from here on to check the expression or activity of YAP as well as kinases involved in Hippo signaling, since these might underlie defects in pancreas development resulting from non-coding genetic mutations or adverse environmental cues. A new path has been opened ahead and we now count with an epigenomic road map to evaluate causes of human pancreas disease that could be linked with its developmental process.
